# Histone Deacetylase Inhibition Restores Retinal Pigment Epithelium Function in Hyperglycemia

**DOI:** 10.1371/journal.pone.0162596

**Published:** 2016-09-12

**Authors:** Danielle Desjardins, Yueying Liu, Craig E. Crosson, Zsolt Ablonczy

**Affiliations:** Department of Ophthalmology, Medical University of South Carolina, Charleston, SC, 29425, United States of America; Indiana University School of Medicine, UNITED STATES

## Abstract

In diabetic individuals, macular edema is a major cause of vision loss. This condition is refractory to insulin therapy and has been attributed to metabolic memory. The retinal pigment epithelium (RPE) is central to maintaining fluid balance in the retina, and this function is compromised by the activation of advanced glycation end-product receptors (RAGE). Here we provide evidence that acute administration of the RAGE agonist, glycated-albumin (gAlb) or vascular endothelial growth factor (VEGF), increased histone deacetylase (HDAC) activity in RPE cells. The administration of the class I/II HDAC inhibitor, trichostatin-A (TSA), suppressed gAlb-induced reductions in RPE transepithelial resistance (*in vitro*) and fluid transport (*in vivo*). Systemic TSA also restored normal RPE fluid transport in rats with subchronic hyperglycemia. Both gAlb and VEGF increased HDAC activity and reduced acetyl-α-tubulin levels. Tubastatin-A, a relatively specific antagonist of HDAC6, inhibited gAlb-induced changes in RPE cell resistance. These data are consistent with the idea that RPE dysfunction following exposure to gAlb, VEGF, or hyperglycemia is associated with increased HDAC6 activity and decreased acetyl-α-tubulin. Therefore, we propose inhibiting HDAC6 in the RPE as a potential therapy for preserving normal fluid homeostasis in the hyperglycemic retina.

## Introduction

Diabetic retinopathy (DR) is a leading cause of blindness in the developed world [1]. As of 2012, diabetes affected 9.3% (29.1 million people) in the United States, of which 19.1% (5.5 million people) were visually impaired [1]. The accumulation of fluid in the diabetic neurosensory retina, broadly termed diabetic macular edema (DME), produces the highest incidence of vision loss (3.8%) [2–6], representing a significant cost to productivity and quality of life. Although glycemic control delays the onset of DR, rates of visual complications (such as DME) increase proportionally with disease duration.

Diabetic macular edema can develop at any point during the course of the disease [7] and can be exacerbated by intensive insulin therapy [8, 9]. It is generally accepted that DME is the result of a breakdown in the two blood retina barriers (BRBs). The inner BRB is formed by the endothelial cells of the retina vasculature, and the outer BRB is formed by the RPE cells. In addition, DME is associated with elevated levels of advanced glycation end-products (AGEs) and VEGF in the vitreous fluid of diabetic patients [10–16]. The resulting development of leaky angiogenic vessels in the inner retina [11] have been the primary focus of most research focused on how hyperglycemia leads to DME. However, more recently our laboratory and others have shown that AGEs and VEGF also target the RPE [11, 17, 18], increasing permeability and diminishing the ability of this tissue to actively remove fluid from the extracellular retinal environment.

Alterations in patterns of protein acetylation are thought to play a pivotal role in the diseases of the blood-brain barrier and HDAC inhibitors have been shown to maintain blood-brain barrier integrity under conditions of pathophysiological stress [19–21]. HDACs regulate protein function and structure by removing the acetyl groups placed on the ε-amino group of lysine by histone acetyl transferases (HATs) [22–24]. There are four classes of HDACs with differing targets and specificities [24]. Class I HDACs (HDAC 1,2,3 and 8) localize to the nucleus and regulate gene expression. Class II HDACs are divided into Class IIa (HDACs 4,5,7, and 9), which shuttle between the cytoplasm and nucleus, and Class IIb (HDACs 6 and 10), which predominantly target substrates in the cytoplasm. Class III HDACs are the NAD+ dependent sirtuins, and HDAC11 constitutes class IV by itself [25]. In diabetic models, HDAC inhibitors have been shown to restore glycemic control in the liver [26], and inner retinal vessels exhibit elevated levels of class I HDACs as well as changes in HAT activity [23, 27, 28]. However, the role of protein acetylation in the modulation of RPE function has not received significant attention in the literature.

Current therapies for DME focus on reducing either the production (e.g., pan-retinal photocoagulation) [29, 30] or effect of VEGF via pharmacological interventions to block VEGF or its downstream signaling events (e.g., anti-VEGF agents and steroids [7, 31, 32]) in ocular endothelia. As all these therapies have significant risks and side effects, new pharmacological interventions are actively under investigation. In the current study, we demonstrate that in acute and subchronic models of ocular hyperglycemia, HDAC inhibitors prevent the breakdown of both functions of the RPE relevant to edema development (i.e., passive barrier and active fluid transport). Moreover, the data indicate that the protective ability of HDAC inhibitors is associated with blocking VEGF-induced deacetylation of RPE microtubules.

## Methods

### Tissue culture

ARPE-19 and fhRPE cells were obtained and cultured on permeable membrane filters as described before [33]. Basic cell treatments with albumin and gAlb were identical to those described previously [34]. In addition, for selected experiments, 100 nM TSA (Enzo Life Sciences, Farmingdale, NY) was administered apically 1 h prior to each albumin treatment. TEER was recorded at the time of TSA pre-administration and for 6 h post albumin administration. In experiments using VEGF, 100 μg/mL albumin was co-administered with TSA or vehicle control to act as a carrier protein for TSA.

### Animals

Animal handling was performed in accordance with the ARVO Statement for the Use of Animals in Ophthalmic and Vision Research; and the study protocol was approved by the Animal Care and Use Committee at the Medical University of South Carolina (AR#3254). Animals were housed in the AAALAC-approved MUSC animal facility and were monitored daily for cleanliness, nourishment, and signs of potential pain and distress by trained facility and laboratory staff. Animals exhibiting signs of illness or discomfort were removed from the studies according to the approved protocol (AR#3254). All animals used for the experiments, Dutch-belted rabbits weighing 1.5–2 kg and Brown Norway rats weighing 130–150 g were obtained from Jackson Laboratories (Bar Harbor, ME) and were used according to experimental procedures previously described [34, 35].

Selected rabbits received 3 μg intravitreal TSA (Enzo Life Sciences, Farmingdale, NY) dissolved in 5% dimethyl-sulfoxide (DMSO) co-administered with albumin or glycated albumin. Subretinal bleb experiments were performed following 48 h incubation [34].

The induction of hyperglycemia in rats is previously described [36]. At 8.5 weeks post induction of hyperglycemia using streptozotocin, selected hyperglycemic and control rats were injected intraperitoneally twice a day with TSA (1 mg/mL, 10% DMSO in 0.9% saline) for four days. On the fourth day after the last TSA injection, subretinal bleb experiments were performed and rates of bleb resorption were calculated as previously described [18].

### Immunoblots

Western blots were performed following the determination of total protein in RPE cultures according to previously established methods [34]. Blocked blotting membranes were incubated with monoclonal mouse anti-acetylated-α-tubulin (Santa Cruz, Dallas, TX) or mouse anti-β-actin (Sigma-Aldrich, St Louis, MO) overnight at 4°C. After treatment with HRP-conjugated secondary antibody for two hours and with chemiluminescent reagent (Fisher Scientific, Fair Lawn, NJ), the lanes were visualized with a VersaDoc 5000 imager (Bio-Rad, Hercules, CA). Actin was used as control to avoid any confounding effects that reblotting for total tubulin would cause following a primary blot for acetyl-α-tubulin. Changes in either actin or total tubulin expression were not significantly different in the observed conditions.

### HDAC activity assay

The deacetylase activities of HDAC1, 2, 3, and 6 were measured by assaying enzyme activity using trypsin and the fluorophore-conjugated synthetic substrate, *t*-butyl- acetyl-lysine amino methoxy-coumarin (Boc-Lys(Ac)-AMC; Enzo Life Sciences, Farmingdale, NY), as previously described [37]. Lysates were centrifuged at 20,000*g* for 10 min and the pellet discarded. 3–5 μL of sample were added to standard HDAC buffer (50 mM Tris-Cl pH 8.0, 137 mM NaCl, 2.7 mM KCl, 1 mM MgCl_2_ and 0.1 mg/mL bovine serum albumin) and incubated with the conjugated-fluorophore acetylated lysine substrate Boc-Lys(Ac)-AMC in 96-well non-binding plates (Greiner Bio-one, NC) at room temperature for 2 h. The substrate in this assay is specific to HDAC1, 2, 3 and 6. At the same time, lysates were incubated with tubastatin-A (TubA; 1 μM; Cayman Chemical, Ann Arbor, MI) in HDAC buffer to block HDAC6 activity. TubA is a modified hydroxamic acid that exhibits over 1000-fold selectivity against all HDAC isoforms excluding HDAC8, where it showed approximately 57-fold selectivity [38].

### Statistical analysis

All values represent a mean of at least 6 independent experiments ± SEM. Pairwise data were analyzed using the Student *t* test and were considered statistically significant at *p* < 0.05. Where multiple comparisons were required, results were compared with one-way ANOVA, Bartlett’s post-test (α = 0.05) using Prism 6 software (Graphpad Software, Inc, La Jolla, CA).

## Results

### HDAC inhibition prevents glycated-albumin-induced RPE barrier breakdown

**Fig 1:**
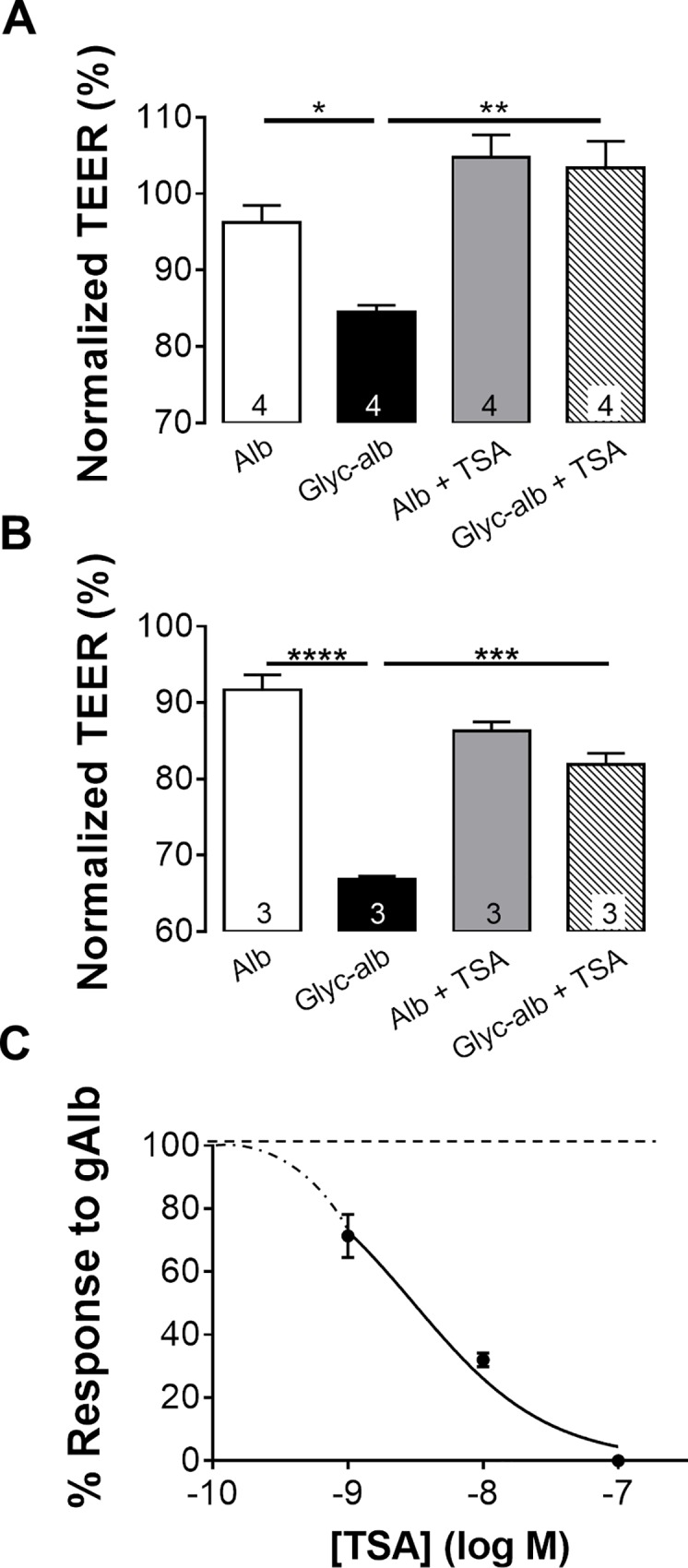
HDAC inhibition blocks Glyc-alb induced reduction in TEER. Administration of 100 μg/mL Glyc-alb with and without TSA pretreatment (1 h) to (A) ARPE 19 cells and (B) hfRPE cells showing the resulting TEER at 6 h post treatment compared to the administration of the same concentration of Alb. (C) Concentration-response curve to TSA determined for ARPE19 cells exposed to 100 μg/mL Glyc-alb. Values represent means ± SE of individual measurements normalized to average TEER at 0 h, analyzed by one-way ANOVA. Column numbers represent *n* for each condition. *p<0.05, **p<0.01, ***p<0.001. Legend: Alb, albumin; Glyc-alb, gAlb, glycated-albumin; TEER, transepithelial electrical resistance; TSA, trichostatin-A.

Baseline TEER measurements for cultures ARPE19 and fhRPE monolayers develop TEER values of 43 ± 5 Ωcm^2^ and 1046 ± 43 Ωcm^2^, respectively [17, 33]. The absolute TEER values in the current study (41 ± 6 Ωcm^2^ and 1032 ± 58 Ωcm^2^ (n ≥ 6)) were not significantly different from these previously reported measurements. To test whether HDAC inhibition can prevent RPE barrier breakdown *in vitro*, monolayers of ARPE-19 and fhRPE cells were treated apically with 100 μg/mL albumin or glycated-albumin. In ARPE-19 cells (**Fig 1A**), the administration of albumin alone did not significantly change the transepithelial electrical resistance (TEER) following 6 h incubation (normalized to the TEER measured at pre-treatment); however, a 12% decrease in TEER was measured when exposed to gAlb for the same amount of time. This effect was completely abrogated by 1 h pretreatment with 100 nM TSA. Pretreatment with TSA alone did not significantly alter the TEER from baseline.

To investigate the role of HDACs in a physiologically more precise RPE model, fetal human RPE (fhRPE) monolayers were treated with 100 μg/mL albumin or glycated-albumin in the presence of TSA (100 nM). The administration of gAlb induced a 25% drop in TEER observed after 6 hours. Again, no statistical reduction in TEER was measured following albumin administration. Co-treatment with 100 nM TSA partially, and significantly (*p < 0*.*001*) suppressed the effect of gAlb in fhRPE cells (**Fig 1B**). Treatment with TSA alone or in combination with albumin did not appreciably alter TEER. These experiments established that class I/II HDAC inhibition in the RPE prevented barrier breakdown.

To determine if the TSA response was concentration dependent, ARPE-19 monolayers were pretreated for 1 h with TSA (0.1–100 nM) followed by gAlb (100 μg/mL) administration. **Fig 1C** shows TEER measurements at 6 h after gAlb exposure, normalized to baseline TEER. The response was concentration dependent with a calculated LogIC_50_ of -8.51 ± 0.049, corresponding to an IC_50_ of 3.06 nM (Hill coefficient = -0.88 ± 0.0.7; R^2^ = 0.97). These data are consistent with a classical binding process mediated by a single target.

### HDAC inhibition maintains RPE fluid transport against glycated-albumin

**Fig 2:**
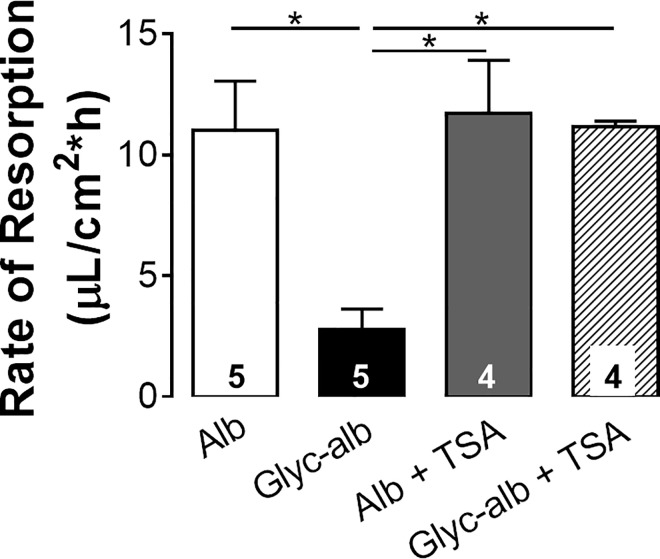
Intravitreal administration of glycated albumin decreases in vivo rates of resorption in rabbits. Two days after intravitreal injection of albumin or glyc-alb (1 mg) coadministered with TSA (3 μg) or vehicle control (dmso, 5%), subretinal bleb resorption rates were measured. Rates are expressed as μL/cm^2^*h. Number of animals in each group are indicated in the columns. *p<0.05, ANOVA. Legend: Alb, albumin; Glyc-alb, glycated-albumin; TSA, trichostatin-A.

To assess if HDAC inhibition can prevent the acute gAlb-induced reduction in RPE fluid resorption, rabbits were injected intravitreally with 1 mg albumin or gAlb. The animals rested and recovered for 48 hours and then a subretinal saline bleb was created. In albumin-treated rabbits, the average resorption rate was 11.01 ± 4.6 μL/cm^2^*h (**Fig 2**). In rabbits treated with gAlb, the average rate of resorption was reduced to 2.79 ± 1.7 μL/cm^2^*h, which was significantly less than the rate measured in the control albumin-treated eyes. Co-administration of TSA (3 μg) to animals receiving albumin or gAlb resulted in resorption rates of 11.71 ± 4.9 and 11.17 ± 0.45 μL/cm^2^*h, respectively. Thus, resorption rates in eyes receiving TSA were not significantly different from control eyes receiving albumin alone.

### HDAC inhibition rescues RPE fluid transport in hyperglycemia

**Fig 3:**
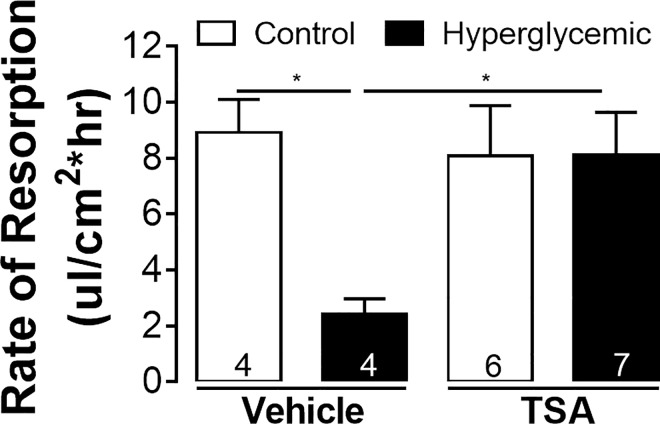
TSA restores resorption rates after 9 weeks of hyperglycemia. 8 week old brown Norway rats were injected with STZ or citrate buffer. After 8.5 weeks of hyperglycemia, rats were treated intraperitoneally, twice daily with 2.5 mg/kg TSA, after which subretinal PBS blebs were placed and resorption measured. Number of animals in each group are indicated in the columns. *p<0.05, one-way ANOVA. Legend: TSA, trichostatin-A.

To better replicate potential RPE dysfunction in diabetes, RPE fluid resorption was evaluated in normal and hyperglycemic rats. After 8.5 weeks of STZ-induced hyperglycemia, hyperglycemic or control rats were treated twice-a-day for 4 days with TSA (2.5 mg/kg; intraperitoneal) and RPE fluid resorption evaluated the following day. **Fig 3**shows that in sham-treated euglycemic controls the rate of fluid resorption was 8.92 ± 1.19 μL/cm^2^*h. Following 9 weeks of hyperglycemia, resorption rates were significantly lower than control values (2.43 ± 0.55 μL/cm^2^*h). However, the brief TSA treatment regime at week 8.5 restored rates to 8.1 ± 1.5 μL/cm^2^*h, not significantly different from euglycemic levels.

### HDAC activity is induced by glycated-albumin

**Fig 4:**
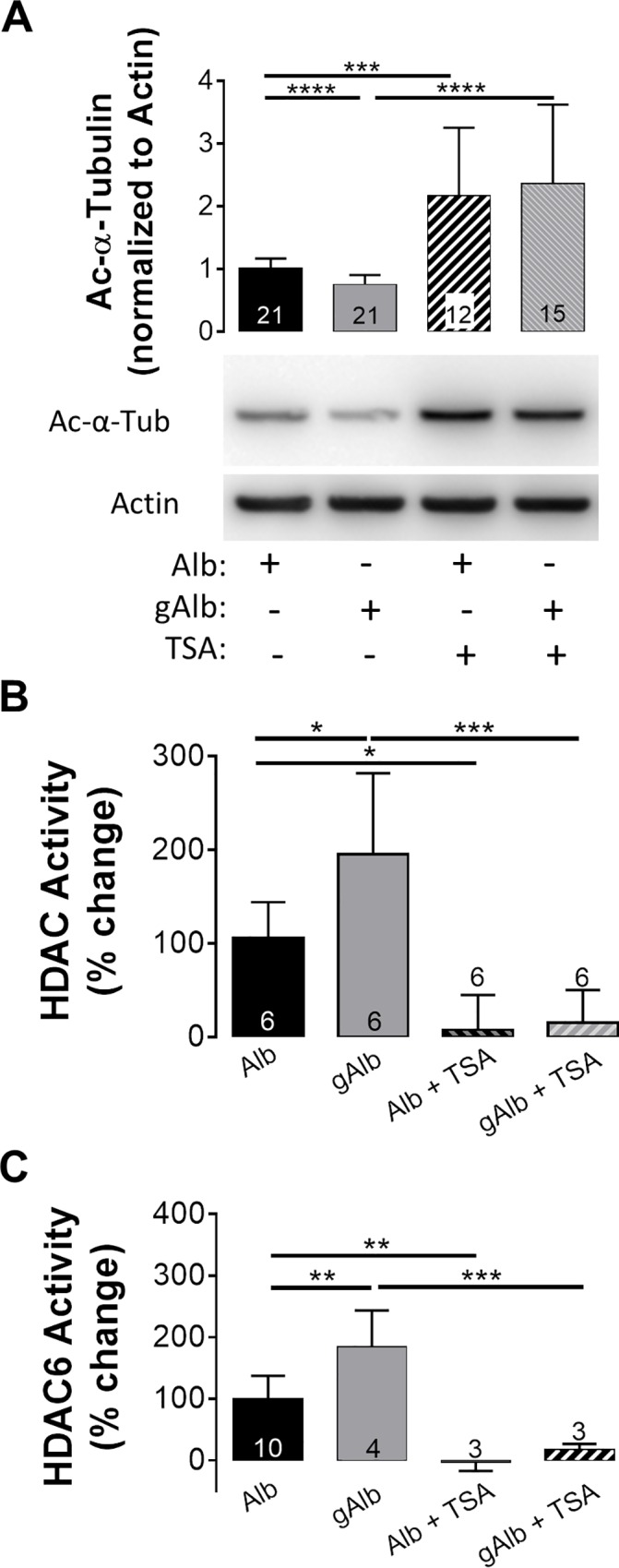
HDAC inhibition blocks Glyc-Alb induced activation of HDACs. (A) ARPE19 cells showed a significant decrease in Ac-α-tubulin at 6 h post treatment of Glyc-alb (100 μg/mL) compared to administration of the same concentration of Alb. ARPE19 cells showed an increase in HDAC1/2/3/6 activity (B) and HDAC6 activity (C) at 6 h post treatment with Glyc-alb (100 μg/mL) compared to the administration of the same concentration of Alb. 1 h pre-treatment with 100 nM TSA (a pan-HDAC inhibitor) prevented the effect of Glyc-Alb. Values represent means ± SE of individual measurements normalized to average TEER at 0 h, analyzed by Student T-test. Column numbers represent *n* for each condition. **p<0.01, ***p<0.001. Legend: Ac-α-tubulin, Ac-α-tub, acetyl-α-tubulin; Alb, albumin; Glyc-alb, glycated-albumin; HDAC, histone deacetylase; TSA, trichostatin-A.

Potentially, class I/II HDAC inhibition with TSA has numerous targets. To determine gAlb-induced changes in protein acetylation, lysates of ARPE-19 monolayers treated with gAlb in the presence or absence of TSA (100 nM) were examined for levels of acetyl-α-tubulin using immunoblotting (**Fig 4A**). We found that the addition of 100 μg/mL gAlb reduced acetyl-α-tubulin levels to 0.66 ± 0.11-fold of normal (a 34% decrease), when compared to monolayers treated with the same concentration of albumin. Pretreatment of monolayers with TSA significantly increased acetyl-α-tubulin levels over two-fold above baseline levels in monolayers that received either albumin or gAlb alone.

To link the decrease in α-tubulin acetylation to HDACs, an HDAC activity assay (using a substrate for HDAC1, 2, 3, and 6) was performed in the same lysates. **Fig 4B** shows that in ARPE-19 monolayers, HDAC activity was significantly increased 195 ± 8% following 6 h incubation with gAlb when compared to normal albumin. In both albumin and gAlb treated monolayers, 1 h pretreatment with 100 nM TSA reduced HDAC activity to 15 ± 14%, which was below baseline.

To determine specific activity of HDAC6 alone, HDAC activity was calculated in wells treated with TubA and the resulting value was subtracted from total HDAC activity in TubA-naïve wells. **Fig 4C** shows that in ARPE-19 monolayers, HDAC6 activity was significantly increased 184 ± 29.8% following 6 h incubation with gAlb when compared to normal albumin. In both albumin and gAlb treated monolayers, 1 h pretreatment with 100 nM TSA reduced HDAC6 activity. Activity was -2.9 ± 8.2% for albumin and TSA and 17.4 ± 5.3% for gAlb with TSA, both below baseline levels.

### VEGF signaling induces HDAC activation

**Fig 5:**
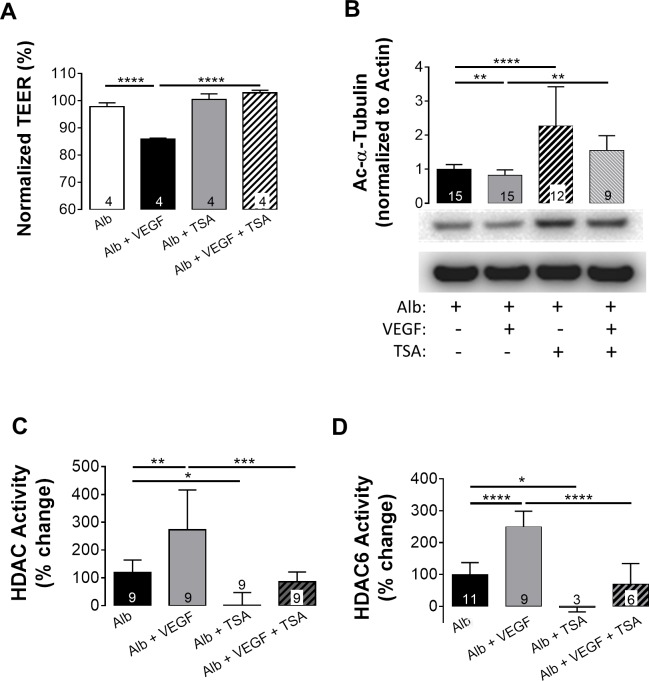
VEGF induced HDAC activity is blocked by Trichostatin-A. (A) Administration of 1 ng/mL VEGF to ARPE 19 cells decreased TEER significantly; reduction in TEER was blocked by TSA (100 nM) pretreatment. (B) VEGF treated ARPE19 cells were lysed after 6 h and blotted for Ac-α-tubulin; β-actin was used as an internal control. 1 h pre-treatment with 100 nM TSA (a pan-HDAC inhibitor) prevented the effect of Glyc-alb, increasing Ac-α-tubulin well above basal levels seen with Alb alone. ARPE19 cells showed an increase in HDAC1/2/3/6 activity (C) and HDAC6 activity (D) at 6 h post treatment with VEGF (1 ng/mL) compared to the administration of the same concentration of Alb; this increase was blocked with TSA pretreatment. Values represent means ± SE of individual measurements normalized to average TEER at 0 h, analyzed by Student T-test. **p<0.01, ***p<0.001. Column numbers represent *n* for each condition. Legend: Ac-α-tubulin, acetyl-α-tubulin; Alb, albumin; HDAC, histone deacetylase; TEER, transepithelial electrical resistance; TSA, trichostatin-A; VEGF, vascular endothelial growth factor.

To determine whether the activation of HDACs and the deacetylation of α-tubulin occur upstream or downstream of VEGF, ARPE-19 cells were administered 2 ng/mL VEGF with or without 1 h TSA (100 nM) pretreatment. As expected from our previous studies [17, 39], following 6 h incubation with VEGF, TEER was significantly decreased. In serum-free media, pretreatment with TSA did not inhibit the VEGF response, potentially indicating that HDACs are activated upstream of VEGF release (data not shown). Albumin has previously been utilized as a carrier for hydrophobic compounds [40]. Therefore, we repeated the VEGF studies with 100 μg/mL albumin in each condition. Compared to albumin alone, administration of VEGF lowered TEER by 18% when following 1 h pretreatment with albumin (**Fig 5A**). However, this decrease was completely abrogated by co-administering TSA with the albumin. Therefore, in further experiments using TSA under serum-free conditions, albumin was always supplemented.

To reinforce that HDAC activation is downstream of VEGF, lysates from ARPE-19 monolayers (treated with 2 ng/mL VEGF in the presence or absence of TSA) were examined for levels of acetyl-α-tubulin by immunoblotting. Compared to albumin, acetyl-α-tubulin was significantly decreased following VEGF stimulation (0.82 ± 0.12 fold) and this response was blocked by 1 h pretreatment with TSA (**Fig 5B**), restoring levels to 1.64 ± 0.36 fold of the albumin control. Analysis of HDAC activity demonstrated that total HDAC activity increased by 273 ± 41%, after 6 h incubation with VEGF when compared to 100 μg/mL albumin alone (**Fig 5C**). Again, pretreatment with TSA decreased HDAC activity of VEGF exposed cells to below baseline levels (85 ± 12%).

To determine the specific activity of HDAC6 in response to VEGF, a HDAC activity assay similar to **Fig 4C**, was performed. **Fig 5D** shows that in ARPE-19 monolayers, HDAC6 activity was significantly increased 249 ± 16.4% following 6 h incubation with VEGF when compared to albumin alone. In both albumin and VEGF treated monolayers, 1 h pretreatment with 100 nM TSA reduced HDAC6 activity. Activity was -2.9 ± 8.2% for albumin and TSA and 69.1 ± 26.6% for VEGF with TSA, both below baseline levels.

### HDAC6 activation mediates RPE dysfunction

**Fig 6:**
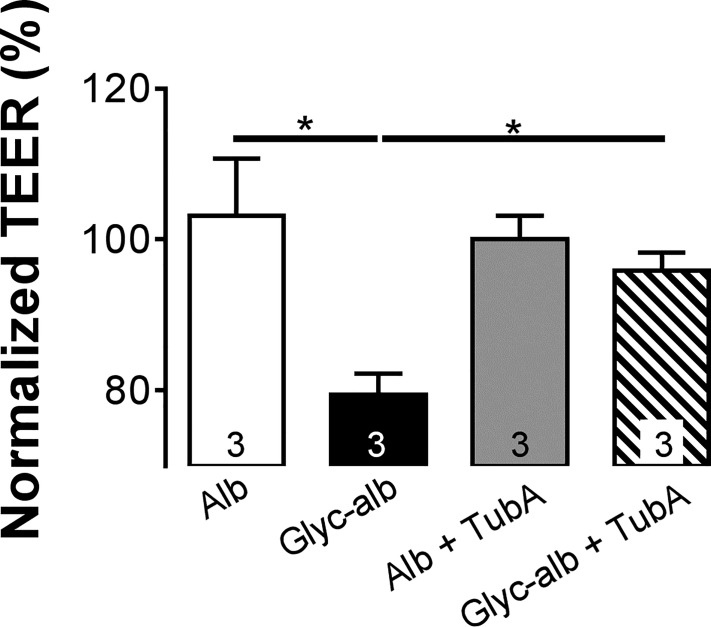
HDAC6 inhibition blocks effect of Glyc-Alb. Pretreatment of ARPE19 cells with 1 μM TubA (an HDAC6 specific inhibitor) for 1 h prevented the reduction in TEER seen with Glyc-Alb measured at 6 h post treatment. Values represent means ± SE of individual measurements normalized to average TEER at 0 h, analyzed by ANOVA. Column numbers represent *n* for each condition. *p<0.05. Legend: Alb, albumin; Glyc-alb, glycated-albumin; TEER, transepithelial electrical resistance; TubA, tubastatin-A.

The enzyme responsible for α-tubulin deacetylation is HDAC6 [41–43]. To determine if HDAC6 is involved in the response to gAlb, the relatively selective HDAC6 inhibitor, TubA (1 μM), was administered to ARPE-19 monolayers. As shown in **Fig 6**, pretreating ARPE19 monolayers with TubA suppressed the response to gAlb (100 μg/mL; 6 h). Again, neither albumin nor TubA administered alone significantly altered TEER.

## Discussion

Along with the increasing incidence of diabetes in developed nations, prevalence of DR has increased in recent years [6]. Current surgical or pharmacological therapies of DME carry risks of significant side-effects. Hence, understanding the pathophysiological mechanisms that contribute to the development of DME should lead to new treatment paradigms and a significant improvement in the quality of life for affected patients. As DME does not regress with the restoration of glycemic control, it is hypothesized that stable post-translational and epigenetic modifications are involved with the pathogenesis of the condition [35, 44, 45]. Herein, we investigated the role of HDACs in the function of the outer BRB, the RPE, essential for the active removal of fluid from the neurosensory retina [46, 47].

The role of HDACs was studied in acute and subchronic models of the diabetic eye, where we have previously provided evidence of significant changes in RPE function [18, 33, 34, 36]. In the acute *in vitro* model, barrier function was measured in human RPE cultures, ARPE-19 cells, and the functionally more accurate fhRPE cells. In both of these cell types, class I/II HDAC inhibition by TSA prevented the breakdown in barrier function induced by gAlb in the apical media (**Fig 1**). *In vivo*, we found that the acute administration of gAlb suppressed subretinal fluid resorption and that co-administration of TSA blocked this response (**Fig 2**). As these acute models do not fully capture the complexity of ocular diabetes, we have also investigated the effect of TSA in hyperglycemic rats. Importantly, systemic TSA administration (four days, twice daily) after 8.5 weeks of hyperglycemia was also able to restore RPE function (**Fig 3**). Thus, the RPE dysfunction associated with acute RAGE activation and subchronic hyperglycemia is prevented by TSA administration. Taken together, these studies suggest a high clinical potential, since HDAC inhibition is beneficial not only preventatively, but also as restorative treatment of both barrier and resorptive RPE functions relevant to fluid accumulation in the retina.

In all three of the above models, we have previously demonstrated that damage to the function of the RPE is at least in part mediated by VEGF [18, 36, 39]. Therefore, to determine if TSA targets cellular events up or downstream of VEGF, the effect of TSA on VEGF-induced changes in RPE function were evaluated. We found that TSA prevented the VEGF-induced breakdown of the RPE barrier (**Fig 5**). However, TSA was only efficacious in the presence of albumin. This is consistent with the idea that albumin serves as a potent carrier protein for TSA. Studies using TSA are historically difficult to reproduce, so much so that laboratories often prefer other similar HDAC inhibitors. Our experiments show that a key aspect of the variability in results with TSA is the presence or absence of a proper carrier protein. Our data provided new evidence that in terms of RPE barrier breakdown, HDAC inhibition targets cellular events downstream of VEGF receptor signaling [34].

HDACs were first discovered as histone modifiers [24]. Thus, it could be argued that the response of the damaged RPE to TSA is due to epigenetic regulation. However, the short time-scale of TSA action in the acute models (especially *in vitro*) lead us to think that changes in expression profiles are not responsible for the loss of barrier function following exposure to gAlb or VEGF. Indeed, it is well known that HDACs also target non-histone proteins [48–50]. The administration of TSA improved tight junction stability in various endothelia [51, 52] and epithelial tissues [53]. In epithelia, the stability of the tight junctions is regulated by interactions with a lateral microtubule network [50], which depends on the acetylation levels of α-tubulin [54–56]. Although it is feasible that TSA can stabilize the same structures, microtubule instability in the context of RPE barrier breakdown has not been previously investigated.

The results in **Figs 4**and **5**provided convincing evidence that RAGE and VEGF receptor agonists lead to a significant depletion of cytosolic acetyl-α-tubulin. It is important to note here, that the levels of acetyl-α-tubulin (cellular structure) correlated remarkably well with TEER (barrier function). Confirming these observations, we have also found that tubulin acetylation is inversely related to HDAC activity. These data demonstrated that decreased α-tubulin acetylation is a consequence of an increase in HDAC activity rather than being a result of decreased HAT activity. Deacetylation of α-tubulin is associated with HDAC6 activity [50, 56], which was clearly increased in response to both gAlb and VEGF (**Figs 4C** and **5D**, respectively). Moreover, functional RPE responses to the HDAC6 antagonist, TubA (**Fig 6**), were essentially identical with those of the class I/II HDAC inhibitor, TSA. Taken together these results provide convincing evidence that RAGE and VEGF stimulate HDAC6 activity in the RPE.

The rapid changes in RPE barrier function induced by gAlb and VEGF argue that the increase in HDAC6 activity directly alters junctional proteins or systems that influence their stability. Although HDAC6 can target various proteins (e.g. peroxiredoxins, HSP90, cortactin, ubiquitin), α-tubulin represents the most likely target involved in the barrier response. The integrity of epithelial tight junctions are regulated by interactions with a lateral microtubule network [50] and the stability this network depends on the acetylation levels of α-tubulin [54–56]. Separate studies have shown that deacetylated microtubules turn over 3–4 times faster (~5–20 minutes) than acetylated microtubules [57]. Therefore, we hypothesize that a key step in hyperglycemic induced RPE dysfunction is HDAC6 mediated hypoacetylation and eventual destabilization of the microtubule network. Additional studies will be needed to validate this hypothesis and understand the precise role of HDACs in regulating RPE tight junction stability.

Multiple papers in the literature tout the protective effects of HDAC inhibitors in glycemic control [27, 58, 59]. Our experiments have provided new evidence that inhibition of HDACs in acute and subchronic models of ocular hyperglycemia also prevents RPE dysfunction. As these RPE functions are required to maintain normal extracellular fluid balance in the neuroretina, we speculate that HDAC inhibitors will be efficacious in treating DME. Moreover, we provide initial evidence that RPE dysfunction associated with hyperglycemic stress is possibly due to deacetylation of RPE microtubules. A link between microtubule acetylation and tight junction stability in epithelial tissue has been established in the intestinal system [54]. Our study opens the door to exploring the stability and regulation of similar cytoskeletal structures in the RPE, complementing results that hyperglycemia can also induce longer-term changes in expression of relevant receptors, such as VEGF-R2 [60].
